# CrackCLIP: Adapting Vision-Language Models for Weakly Supervised Crack Segmentation

**DOI:** 10.3390/e27020127

**Published:** 2025-01-25

**Authors:** Fengjiao Liang, Qingyong Li, Haomin Yu, Wen Wang

**Affiliations:** 1 Key Laboratory of Big Data & Artificial Intelligence in Transportation (Beijing Jiaotong University), Ministry of Education, Beijing 100044, China; liangfj@bjtu.edu.cn (F.L.); liqy@bjtu.edu.cn (Q.L.); 2 Frontiers Science Center for Smart High-Speed Railway System, Beijing Jiaotong University, Beijing 100044, China; 3 Department of Computer Sicence, Aalborg University, 9200 Aalborg, Denmark; haominyu@cs.aau.dk

**Keywords:** weakly supervised crack segmentation, vision-language model, Contrastive Language–Image Pre-Training

## Abstract

Weakly supervised crack segmentation aims to create pixel-level crack masks with minimal human annotation, which often only differentiate between crack and normal no-crack patches. This task is crucial for assessing structural integrity and safety in real-world industrial applications, where manually labeling the location of cracks at the pixel level is both labor-intensive and impractical. Addressing the challenges of labeling uncertainty, this paper presents CrackCLIP, a novel approach that leverages language prompts to augment the semantic context and employs the Contrastive Language–Image Pre-Training (CLIP) model to enhance weakly supervised crack segmentation. Initially, a gradient-based class activation map is used to generate pixel-level coarse pseudo-labels from a trained crack patch classifier. The estimated coarse pseudo-labels are utilized to fine-tune additional linear adapters, which are integrated into the frozen image encoders of CLIP to adapt the CLIP model to the specialized task of crack segmentation. Moreover, specific textual prompts are crafted for crack characteristics, which are input into the frozen text encoder of CLIP to extract features encapsulating the semantic essence of the cracks. The final crack segmentation is determined by comparing the similarity between text prompt features and visual patch token features. Comparative experiments on the Crack500, CFD, and DeepCrack datasets demonstrate that the proposed framework outperforms existing weakly supervised crack segmentation methods, and the pre-trained vision-language model exhibits strong potential for crack feature learning, thereby enhancing the overall performance and generalization capabilities of the proposed framework.

## 1. Introduction

Crack segmentation is a specialized application within the field of semantic segmentation, aimed at generating binary masks at the pixel level to identify and outline cracks [1,2,3,4]. This technique is essential for various critical applications, including crack detection in pavements [5,6,7], crack extraction from concrete surfaces [8], road pattern recognition from aerial imagery [9], and blood vessel segmentation in medical diagnostics [10].

Visually, cracks manifest as linear topologies with higher pixel intensity relative to the surrounding background pixels. However, they often exhibit poor continuity and low contrast against the background due to noise interference [11,12,13]. Over the past few years, deep learning-based models have emerged as a leading approach for crack detection, significantly enhancing detection capabilities across diverse scenarios [14]. However, these models heavily rely on extensive manual annotated datasets, which necessitate laborious pixel-level labeling, particularly for small and intricate cracks [15].

To mitigate the reliance on extensive manual annotations, weakly supervised learning methods are proposed for crack image segmentation [15,16,17,18,19]. These methods typically employ binary labels to annotate image patches as either containing cracks or not. Currently, two-stage weakly supervised crack segmentation methods, which include the generation of crack pixel pseudo-labels and the training of segmentation models, achieve superior performance. The methods [15,16] focus on enhancing the accuracy of pseudo-labels through various post-processing operations to refine the initial pixel-level pseudo-labels. Moreover, the approaches [17,18,19] emphasize continuously optimizing the reliability of pseudo-labels during the training process of the segmentation network. Nevertheless, the methods for generating pseudo-labels using only such binary annotations are insufficient to address the significant challenges arising from complex topological structures, irregular crack edges, and low-contrast backgrounds.

Beyond the binary annotation of image patches, we propose that incorporating textual language can introduce additional contextual information. By providing generalized descriptions of the inherent topological features of cracks, textual language not only captures the unique morphological characteristics of cracks but also enhances their generalization across different scenarios. Additionally, by providing a detailed description of the differences between cracks and the surrounding normal background, the characteristics of cracks in images can be highlighted more clearly, thereby significantly improving the accuracy and reliability of crack detection. To leverage textual descriptions to provide additional supervisory information for the crack segmentation task, the Contrastive Language–Image Pre-training (CLIP) [20] model is introduced to jointly train crack images and their corresponding text representations through contrastive learning. This approach enhances the model’s ability to understand and identify crack features. However, CLIP primarily focuses on global alignment between images and text rather than fine-grained pixel-level alignment [21].

In this paper, we propose a weakly supervised crack segmentation method called CrackCLIP for adapting vision-language models. This method leverages the generalization ability of CLIP, a vision-language model, to learn expressive representations that capture broad concepts. The proposed model comprises two main phases: pixel-level pseudo-label generation and CLIP-based crack segmentation. For the pixel-level pseudo-label generation phase, a patch-based crack classifier is trained to generate crack pixel pseudo-labels using Gradient-based Class Activation Mapping (Grad-CAM) [22]. For the crack segmentation phase, the CLIP-based vision-language model is employed to align the crack pixels with crack compositional text prompts, achieving precise crack pixel segmentation. The frozen CLIP model provides a robust foundation for feature extraction, while the additional linear layers are fine-tuned using the generated crack pixel-level pseudo-labels to ensure that the model effectively captures the specific characteristics of crack images. Finally, the crack text features are aligned with the features of the crack image patches to achieve accurate crack image segmentation. To summarize, our main contributions are as follows:

(1) We propose a CLIP-based model, CrackCLIP, for weakly supervised crack image segmentation. The model leverages a pre-trained CLIP with frozen parameters to extract features from both text prompts and crack images, thereby enhancing its generalization capability.

(2) Additional linear layers are introduced to adaptively train the model on crack images, achieving alignment between crack pixels and text prompts. Furthermore, textual prompts for cracks are designed based on epistemic and topological features, enabling the text encoder to capture rich semantic information and improve generalization across diverse crack scenarios.

(3) The proposed weakly supervised method achieves competitive results on the Crack500 [23], CFD [24], and DeepCrack [6] datasets. Notably, the method demonstrates excellent generalization capabilities when trained on the Crack500 training set and tested on the Crack500 testing set, as well as on the CFD and DeepCrack datasets.

## 2. Related Work

### 2.1. Weakly Supervised Crack Segmentation Methods

In recent years, the field of automatic crack detection has witnessed significant advancements, particularly in the realm of weakly supervised learning for crack image segmentation [25,26,27,28]. Within this domain, image-level labels are predominantly utilized in weakly supervised segmentation approaches.

The prevailing methodologies, which are based on image-level labels, typically adopt a two-stage framework encompassing the derivation of crack pixel-level pseudo-labels and the subsequent training of segmentation models. In the derivation of pseudo-labels, a common approach is to employ image-level labels to train classifiers for crack detection, which then generate class activation maps as preliminary crack pixel-level pseudo-labels [29]. König et al. [15] introduced an innovative thresholding technique to refine these initial pseudo-labels, thereby enhancing the precision of crack segmentation models. While such classifiers provide a coarse yet effective localization of cracks and mitigate background noise, they may fail to capture subtle crack details. Dong et al. [16] advanced this approach by integrating conditional random fields to post-process the initial pseudo-labels, thereby improving the quality of pixel-level pseudo-labels. Wang et al. [30] proposed Crack-CAM, a pixel-level weakly supervised segmentation method that leverages clustering within Convolutional Neural Networks (CNNs) to accentuate crack features and elevate the fidelity of pseudo-labels. These methodologies are primarily aimed at enhancing the quality of crack pixel pseudo-labels.

Nevertheless, existing weakly supervised segmentation techniques have also focused on optimizing the training process of segmentation models. Al-Huda et al. [18] proposed a multi-scale class activation map approach to enhance the completeness of initial pseudo-labels and introduced an Incremental Annotation Refinement module to progressively improve these pseudo-labels. In another contribution, Al-Huda et al. [17] combined class activation maps from CNN classifiers with features extracted by the encoder in the segmentation model, feeding the amalgamated features into the decoder to bolster crack segmentation quality.

Similar to the methods above, our proposed method leverages the advantage of Grad-CAM in generating initial pseudo-labels, effectively providing a coarse representation of crack regions within an image. In contrast, the initial pseudo-labels are enhanced with semantic descriptions of the crack appearance in natural language. By integrating a vision-language model, we capture the intricate relationships between the visual features of cracks and the corresponding semantic features in the text. It leverages the robust representational capabilities of large-scale, pre-trained vision-language frameworks, which have demonstrated exceptional performance in understanding and processing multi-modal data. Our dual-modal strategy extends beyond traditional crack detection by not only enriching the feature space for crack detection but also significantly improving the segmentation accuracy. This is achieved by offering a more holistic understanding of the crack context, which is crucial for precision in image segmentation tasks. Consequently, this enhancement leads to a more accurate segmentation of cracks in images.

### 2.2. Vision-Language Modeling Methods

Recent advancements in large pre-trained vision-language models have been notably successful, with Contrastive Language–Image Pre-Training (CLIP) [20] standing out for its ability to model the relevance between images and text. This model enhances understanding by aligning visual content with textual descriptions.

This model has been instrumental in image segmentation, particularly in the Reference Image Segmentation (RIS) approach, which uses textual prompts to identify and segment objects. Liu et al. [31] introduced a weakly supervised RIS method leveraging CLIP, employing a bilateral prompt strategy that includes target and background text prompts. This strategy effectively bridges the domain gap between visual and linguistic features, enabling comprehensive target activation for accurate pixel-level pseudo-labeling. Rao et al. [32] proposed a dense prediction framework that transforms the image–text problem in CLIP into a pixel–text problem. By utilizing pixel–text scores, their model guides dense predictions while enhancing vision-language alignment through contextual image information.

In image anomaly segmentation, CLIP leverages natural language to address the paucity of diverse anomaly samples and precise annotations. Jeong et al. [33] presented WinCLIP, a few-shot/zero-shot anomaly detection approach that employs a multi-scale window-based CLIP model to pinpoint anomaly locations based on textual descriptions. Chen et al. [34] proposed a zero-shot anomaly detection model based on CLIP, which incorporates additional linear layers within the image encoder to map image features to a joint embedding space for anomaly detection.

Inspired by the methods above, this paper presents a method to integrate CLIP into the task of weakly supervised crack segmentation. To adapt the CLIP model, which is pre-trained on natural images, to the specific characteristics of industrial crack images, we devised a strategy incorporating customized textual prompts for cracks and a linear adapter module. This linear adapter is integrated into the frozen image encoder of CLIP, allowing us to fine-tune the adapter using pseudo-labels. This refinement enables CLIP to better adapt to the distinct features of crack images, enhancing segmentation performance and accuracy.

## 3. Methodology

### 3.1. Approach Overview

**Figure 1:**
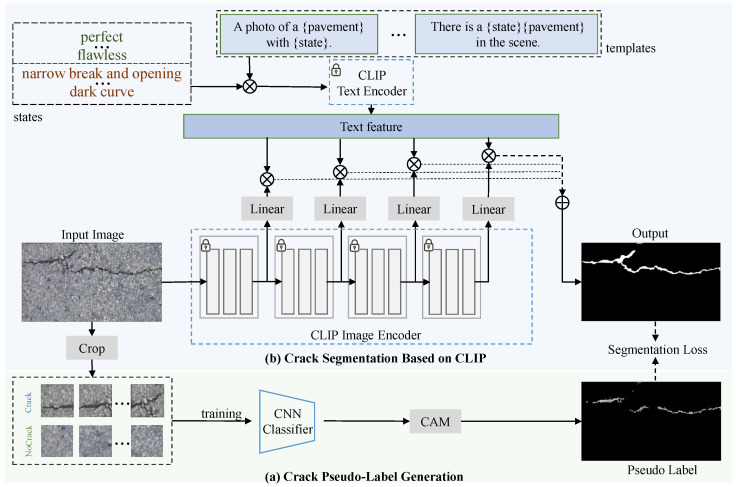
Overview of the proposed CrackCLIP framework. (**a**) Crack pixel-level pseudo-label generation. (**b**) CLIP-based crack segmentation. Phase (**b**) includes a crack image encoder and a crack text encoder. The crack text prompt features are aligned with the crack image patch token features to enable pixel-level crack prediction.

We propose CrackCLIP, a novel adapting visual-language model specifically designed for the task of weakly supervised crack segmentation. Our approach, as illustrated in Figure 1, is designed to integrate pixel-level pseudo-labels with textual prompts to enhance the segmentation of cracks. The framework is divided into two main phases: (a) pixel-level pseudo-label generation and (b) CLIP-based crack segmentation.

The first phase, depicted in Figure 1a, is designed to generate the initial pixel-level pseudo-labels. With patch-level labels indicating the presence or absence of cracks, Gradient-based Class Activation Mapping (Grad-CAM) is employed to highlight potential crack regions. Specifically, the process begins with a CNN classifier that is trained to recognize cracks within image patches. By computing the gradients of the classifier’s output with respect to the input image and weighting them by the importance of each feature map, Grad-CAM produces a class activation map. This map highlights areas crucial for crack identification and is up-scaled to the original image size to provide a preliminary segmentation.

The CLIP-based crack segmentation process, shown in Figure 1b, is comprised of two key branches: a text encoder and an image encoder. The text encoder processes textual prompts that describe the state of the pavement, such as “perfect”, “flawless”, “narrow break and opening”, and “dark curve”. These textual descriptions are designed to guide the model in understanding the context of the cracks and overcome the insufficiency and uncertainty of the weak supervision. The image encoder of CLIP is employed to extract visual features from the input image and is frozen without further fine-tuning. The linear adapter modules, as indicated in the diagram, are added to the image encoder to adapt to the specific features of crack images. These linear layers are optimized through a segmentation loss function, which fits the model’s output with the initial pixel pseudo-labels, thereby enhancing the precision of the crack segmentation.

### 3.2. Crack Pseudo-Label Generation

Following the Grad-CAM [22], this method generates crack activation maps as initial pixel pseudo-labels for the cracks in the image. A dataset D={(x,y)|y∈{0,1}} of crack image patches, including patches with cracks (y=1) and patches without cracks (y=0), is used to train a patch-based crack classifier. For a given input image *x* and corresponding image-level label *y*, the classification network first embeds *x* into a high-level feature mapping $Z\in R^{C\times H\times W}$, where *C* and H×W denote the number of channels and the spatial dimension, respectively. A global average pooling layer and a 1×1 convolution layer with a learnable matrix $A\in R^{C\times N}$, where *N* denotes the number of classes, are then applied to *Z* to obtain the prediction result $\hat{y}\in \left[0,1\right]$. The cross-entropy loss is used to train the crack classifier.

Given the trained image patch classification network, the high-level feature mapping *Z* is weighted by the parameters *A* to generate the initial crack pixel activation map *M*, which is denoted as:

(1) $$M\left(h,w\right)=A^{T}Z\left(h,w\right),$$

where Z(h,w) represents the feature vector located at position (h,w).

While Grad-CAM effectively identifies key regions for crack detection, it may overlook less prominent crack pixels. Given the elongated nature of cracks, the up-sampling process used to generate the crack activation map can inadvertently activate surrounding pixels, potentially reducing the precision of the pseudo-labels. To address this, our method adopts a post-processing strategy, as detailed in [15,16], which refines the pseudo-labels and accurately delineates fine crack pixels.

### 3.3. Crack Segmentation with Vision-Language Alignment

**Figure 2:**
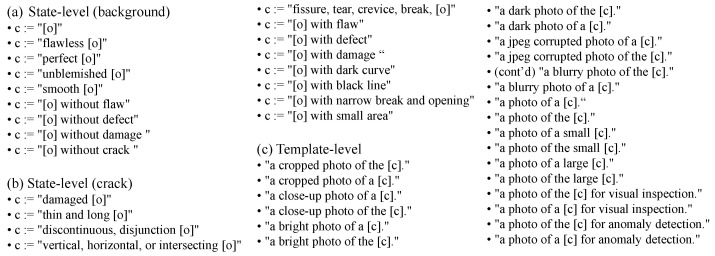
List of crack compositional text prompts. “o” denotes the object in the image.

**Crack Compositional Text Prompts.** This section provides text prompts for crack images, which describe the semantic information of cracks and the background. CrackCLIP defines the two pieces of semantic information of a crack image using a crack compositional text prompting strategy. Specifically, CrackCLIP employs a combination of predefined text templates and state words to describe a given image, rather than freely writing definitions [34]. For example, given an image of a pavement with cracks, the image category is considered to be “pavement”, i.e., the “o” in Figure 2 can be described as “pavement”. The text template is “a photo of a {}”, and the state word for the image is “{} with narrow break and opening”, which together are expressed as: “a photo of a {pavement with narrow break and opening}”. The state words include not only the common states of the target in natural images, such as a normal background being described as “flawless” and cracks being described as “damaged”. They also encompass textual descriptions defined based on the apparent and topological features of the cracks, e.g., cracks are denoted as “narrow break and opening”. Finally, text prompts for cracks provide a list of templates, as shown in Figure 2.

**Crack Segmentation.** The CLIP-based weakly supervised crack segmentation framework is depicted in Figure 1b. This framework is composed of two main branches: a text encoder and an image encoder, which are designed to work in concert to assess the similarity between textual and visual data. In the text feature learning process, the template is combined with the state of the object to construct a crack text description. The text description is encoded by the text encoder of the CLIP model, and the encoded text features are represented as $F_{t}\in R^{N\times C}$, where *N* denotes the number of categories of the image, and *C* denotes the number of channels of the text feature.

The frozen CLIP image encoder is utilized to learn the image feature representation. However, the features are not fully suitable for the crack image scenario and cannot be directly compared with the crack text features using the image features alone. Therefore, this paper follows the approach in [34] by adding additional linear layers to learn specific crack image features and then compares these features with text features. Additionally, the image encoder of CLIP in Figure 1 uses the Vision Transformer (ViT) framework [35], where all feature layers are divided into four stages. A linear layer is applied after each stage to map the output features into the joint embedding space, which is learned based on both the pre-trained data and the crack data. The joint feature $F_{v}^{\prime }$ is represented as:

(2) $$F_{v}^{\prime }=kF_{v}+b,$$

where $F_{v}$ denotes the crack patch token features extracted by the pre-trained CLIP image encoder, and *k* and *b* denote the weights and biases of the linear layer, respectively.

Since the CLIP model is primarily designed for classification tasks, it cannot be directly applied to pixel-level classification. Our objective is to achieve text-to-pixel alignment. Specifically, the CLIP model predicts the probability of a pixel belonging to a crack by computing the similarity between the features of patch tokens and text features. The image encoder’s network architecture is divided into *Q* stages (*Q* is set to 4 in this work), and the joint feature of the patch token is denoted as $F_{v}^{i\prime }$ in the *i*-th stage. To obtain the final crack segmentation map, the proposed CrackCLIP method takes the similarity between the joint image feature $F_{v}^{i\prime }$ and the crack text feature $F_{t}$ as the probability of belonging to the crack. Following the predictive outcomes of crack detection at various stages, the framework employs a multi-scale strategy to effectively integrate shallow and deep features, thereby generating the final crack segmentation image *S*. *S* is represented as:

(3) $$S=\sum_{i=1}^{Q}\mathrm{softmax}\left(F_{v}^{i\prime }{F_{t}}^{T}\right).$$

To ensure that the segmentation image matches the dimensions of the original input image, the method applies an up-sampling process to the final segmentation map.

### 3.4. Segmentation Loss

In the proposed weakly supervised crack segmentation framework, the parameters of both the image encoder and the text encoder based on the CLIP model are frozen, meaning that only the pre-trained model parameters are utilized. However, the additional linear layer is a learnable module that is trained to adapt to the crack image scenario. Therefore, we supervise the crack segmentation map prediction using a linear combination of focal loss [36], dice loss [37], and edge loss [38].

The focal loss addresses class imbalance by reshaping the standard cross-entropy loss, $L_{focal}$, which is defined as:

(4) $$L_{\mathrm{focal}}\left(p_{u}\right)=-\alpha_{u}{\left(1-p_{u}\right)}^{\gamma }\log\left(p_{u}\right),$$

where γ is the focusing parameter, α balances the importance of positive and negative examples, and $p_{u}$ is defined as:

(5) $$p_{u}=\left\{\begin{array}{cc} p & \;\mathrm{if}\;y=1,\\ 1-p & \;\mathrm{otherwise},\; \end{array}\right.$$

where p∈[0,1] denotes the probability in the final crack segmentation map *S* and y∈{0,1} specifies the ground truth.

The dice loss function $L_{dice}$ creates a balance between the crack foreground and background classes by implicitly measuring the overlap between the predicted masks and the ground truth. $L_{dice}$ is defined as:

(6) $$L_{dice}=1-\frac{2\sum_{i}^{N}p_{i}y_{i}}{\sum_{i}^{N}p_{i}^{2}+\sum_{i}^{N}y_{i}^{2}},$$

where *p* and *y* denote the crack prediction probability and the ground truth, respectively.

The edge loss $L_{edge}$ is designed to encourage the segmentation model to produce more accurate predictions in the boundary region. $L_{edge}$ is defined as:

(7) $$L_{edge}=1-\frac{\sum_{i}^{N}p_{i}\cdot E\left(x_{i}\right)}{\sum_{i}^{N}E\left(x_{i}\right)},$$

where *p* denotes the crack prediction probability. $E(x_{i})$ indicates whether the pixel $x_{i}$ belongs to the edge or not, where $E(x_{i})=1$ means that $x_{i}$ is a crack edge pixel and $E(x_{i})=0$ means that $x_{i}$ is not a crack edge pixel.

Finally, the total combined loss $L_{total}$ is denoted as:

(8) $$L_{total}=L_{focal}+L_{dice}+L_{edge}.$$

During the testing process, images are input into the CLIP image encoder of CrackCLIP to extract image features and perform similarity calculations with text features, thereby obtaining the results of crack segmentation. By integrating visual and linguistic information, the model can more accurately identify cracks.

## 4. Results

This section first details the dataset and experimental settings. We then compare our proposed method against other prevalent approaches and conduct ablation studies to evaluate its effectiveness.

### 4.1. Dataset

To demonstrate the performance of the proposed CrackCLIP method, we utilize three challenging and widely used crack image datasets: Crack500 [23], CFD [24], and DeepCrack [6]. In our experiments, the CrackCLIP model is trained using the training set from the Crack500 dataset. The test set comprises the Crack500 testing set, as well as the CFD and DeepCrack datasets, to evaluate the model’s generalization capabilities across diverse datasets.

The Crack500 dataset [23] was collected using a mobile phone at the main campus of Temple University and serves as a pavement cracking dataset. The dataset consists of 1896 crack images used as a training set and 1124 crack images used as a testing set. The resolution of each original image is either 648×484 pixels or 640×360 pixels. To generate crack pixel pseudo-labels for training the CrackCLIP model, a crack image patch dataset is constructed from the Crack500 training set. The Crack500 training dataset is sliced into image patches of 128×128 pixels. Additionally, this work employs rotation and flipping to augment the crack data. As a result, 556,448 images are used to train the crack patch classification network, comprising 238,820 crack images and 317,628 images without cracks. Finally, the original images in the Crack500 training set, along with the generated pseudo-labels of crack pixels, are used to train the crack segmentation network, CrackCLIP.

The CFD dataset [24] was captured using a smartphone, the iPhone 5, in Beijing, China. The images depict road conditions and are taken with a focal length of 4 mm, an aperture of f/2.4, and an exposure time of 1/134 s. The dataset consists of 118 crack images, each with a resolution of 320×480 pixels. The cracks in this dataset are fine, with widths ranging from 1 to 3 mm, and the background contains various types of noise, such as shadows, oil spots, and water stains.

The DeepCrack dataset [6] consists of 537 Red–Green–Blue (RGB) crack images, each with a resolution of 544×384 pixels or 384×544 pixels. The dataset includes crack images with multiple textures, scenes, and scales. In terms of scene distribution, 22% of the cracks belong to asphalt scenes, while the remaining 78% belong to concrete scenes. Regarding texture distribution, 40% of the crack images are classified as rough, 22.4% as stained, and the remaining 37.6% as smooth. Across the entire dataset, 3.54% of the pixels represent cracks, while the remaining 96.46% correspond to background pixels.

### 4.2. Evaluation Metrics

To quantitatively evaluate the detection performance of different models on the crack dataset, we follow the existing crack segmentation methods [5] and evaluate the similarity between model predictions and ground truth using the F1 with the following evaluation strategies: Optimal Dataset Scale (ODS), Optimal Image Scale (OIS), and Average Precision (AP). The ODS denotes that a fixed threshold is selected across the entire dataset to obtain the best F1. The OIS denotes the selection of the threshold corresponding to the optimal F1 for each image to obtain the final F1. The ODS and OIS are denoted as:

(9) $$\begin{array}{cc} & OIS=\frac{1}{N}\sum_{i=1}^{N}\max\left\{F1_{\tau }^{i}:\forall \tau \in \left\{0.01,0.02,\ldots ,0.99\right\}\right\}, \end{array}$$

(10) $$\begin{array}{cc} & \;\;ODS=\max\left\{\left\{\frac{1}{N}\sum_{i=1}^{N}F1_{\tau }^{i}\right\}:\forall \tau \in \left\{0.01,0.02,\ldots ,0.99\right\}\right\}. \end{array}$$

Here τ∈[0,1] represents the selected threshold, and F1 denotes the F1-score. The precision (PR), recall (RE), and F1 are denoted as:

(11) $$\begin{array}{cc} & PR_{\tau }=\frac{TP_{\tau }}{TP_{\tau }+FN_{\tau }}, \end{array}$$

(12) $$\begin{array}{cc} & RE_{\tau }=\frac{TP_{\tau }}{TP_{\tau }+FP_{\tau }}, \end{array}$$

(13) $$\begin{array}{cc} & \;\;F1_{\tau }=2\times \frac{RE_{\tau }\times PR_{\tau }}{RE_{\tau }+PR_{\tau }}. \end{array}$$

Here TP, FP, TN, and FN denote true positive, false positive, true negative, and false negative, respectively. In addition, the average precision (AP) of the model at different recall rates is measured by calculating the area under the precision–recall curve, and AP is denoted as:

(14) $$AP=\sum_{\tau =0.01}^{\tau =1}\frac{1}{T}\left(RE_{\tau }-RE_{\tau -0.01}\right)PR_{\tau },$$

where τ∈[0,1] is the selected threshold, and *T* is set to 100.

### 4.3. Comparison Methods

We compare the performance of CrackCLIP to existing weakly supervised crack segmentation methods. These methods are described as follows:

- Grad-CAM [22]. This method employs Gradient-based Class Activation Mapping to generate pseudo-labels, which can be directly utilized for training crack segmentation models without any post-processing.
- PWSC [16]. This method employs patch-based Grad-CAM combined with conditional random field (CRF) post-processing for the weakly supervised crack segmentation task.
- GPLL [15]. This method generates crack pseudo-labels based on Grad-CAM using localization with a classifier and thresholding to implement the weakly supervised crack segmentation task.
- CAC [19]. This method utilizes crack pseudo-labels with varying confidence levels to co-train a weakly supervised crack segmentation framework.

We utilized crack pixel-level pseudo-labels to train existing crack segmentation backbones and compared the performance of CrackCLIP with other network backbones in a weakly supervised crack segmentation task.

- U-Net [39]. U-Net extends the encoder–decoder architecture by incorporating skip connections, which combine feature maps from the encoder with those from the decoder. This design retains more spatial information and enhances localization accuracy.
- DeepCrack^1^ [6]. DeepCrack^1^ aggregates multi-scale and multi-level features using a fully convolutional neural network to predict crack pixels. A deep supervised network is employed to directly supervise the crack features at each convolutional stage, ensuring robust and accurate feature extraction.
- DeepCrack^2^ [5]. DeepCrack^2^ fuses the convolutional features generated in the encoder and decoder networks based on the SegNet [40] network.
- OED [41]. Based on a fully convolutional U-Net, OED exploits residual connectivity within the convolutional blocks and adds an attention-based gating mechanism between the encoder and decoder parts of the architecture.

### 4.4. Implementation Details

#### 4.4.1. Environment

Our experiments were conducted on a deep learning workstation with Ubuntu 16.04 LTS, equipped with an Nvidia Titan XP GPU (Santa Clara, CA, USA). The framework used for the experiments is PyTorch 1.12 [42].

#### 4.4.2. Experimental Setting

This section details the experimental setup for generating crack pixel-level pseudo-labels as well as the experimental setup for the training and testing phases of the CrackCLIP model. In the crack pixel-level pseudo-label generation process, a ResNet50 [43] network is used to train a binary classification model for crack images. The crack classification model is trained for 10 epochs with a batch size of 16. The initial learning rate is set to $1\times 10^{-3}$, and the learning rate is reduced by a factor of 10 after each epoch. Stochastic Gradient Descent is used as the optimizer with a momentum value of 0.9. The Grad-CAM method [22] is employed to generate pseudo-labels for crack pixels. Subsequently, two post-processing methods are used in this paper to generate fine-grained pseudo-labels: one is threshold segmentation of pseudo-labels based on the literature [15], and the other is the conditional random field for pseudo-labels based on the literature [16].

In the crack segmentation stage of CrackCLIP, our CLIP image encoder utilizes the ViT-L/14 model with an input image resolution of 518×518 pixels. The image encoder consists of a total of 24 layers, which are divided equally into 4 stages, each containing 6 layers. Four additional linear layers are added to extract the crack features. The CLIP model is trained using the Adam optimizer with a fixed learning rate of $1\times 10^{-3}$. The model requires only 3 epochs of training with a batch size of 8. Since an edge-based loss function is employed to preserve the boundaries of the cracks, wider cracks may occur, where background pixels around the cracks are incorrectly classified as cracks. To address this issue, we apply morphological erosion post-processing to the experimental results. Specifically, in our experiments, we use a 3×3 kernel to perform three morphological erosion operations on the crack-segmented images.

### 4.5. Comparison with State-of-the-Art Methods

**Figure 3:**
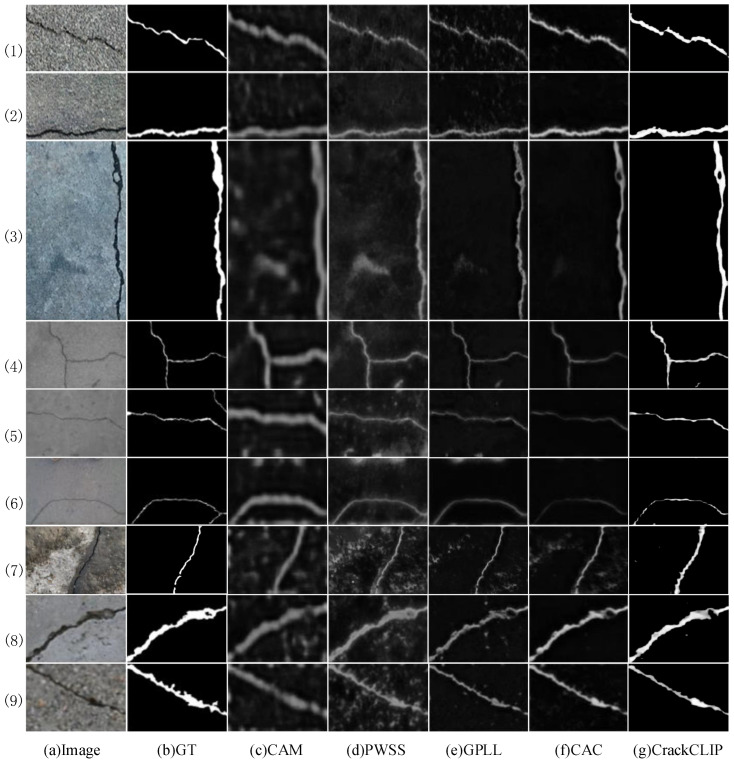
Visualization of crack prediction results for different WSCS methods. Rows (1)–(3) are from the Crack500 testing set, rows (4)–(6) are from the CFD dataset, and rows (7)–(9) are from the DeepCrack dataset.

Table 1 demonstrates the performance of CrackCLIP compared to other weakly supervised crack segmentation (WSCS) methods on the Crack500, CFD, and DeepCrack datasets. To evaluate the accuracy and generalization of CrackCLIP, its performance is compared with several existing weakly supervised crack segmentation methods, including Grad-CAM [22], PWSC [16], GPLL [15], and CAC [41]. The results show that CrackCLIP performs well on the Crack500 dataset, achieving an OIS of 61.31%, an ODS of 68.58%, and an AP of 59.33%. To further validate the generalization of our proposed model, CrackCLIP, and the compared methods, are each trained on the Crack500 training set and subsequently tested on the CFD and DeepCrack datasets. On the CFD dataset, CrackCLIP achieves an OIS of 40.80%, an ODS of 41.74%, and an AP of 31.81%. As illustrated in Figure 3, cracks in the CFD dataset are typically very thin and exhibit much lower contrast with the background compared to those in the training set (Crack500). This significant difference in crack appearance makes generalization considerably more challenging. CrackCLIP leverages a large visual-language model augmented with crack text descriptions. By improving model generalization through semantic mining of crack categories, this approach demonstrates clear advantages over all comparative methods. Specifically, compared to the previous best method, CAC, CrackCLIP shows substantial improvements: 17.49% in OIS, 10.19% in ODS, and 13.26% in AP. On the DeepCrack dataset, CrackCLIP achieves an OIS of 68.26%, an ODS of 73.29%, and an AP of 68.82%, outperforming most other methods in these tests. The DeepCrack dataset exhibits substantial similarity to Crack500, thereby diminishing the apparent generalization advantage of CrackCLIP. Additionally, this dataset includes a significant amount of noise that closely resembles crack features semantically, which heightens the risk of false detections. As a result, while CrackCLIP generally yields superior performance compared to most benchmark methods, it does not exceed CAC, which has undergone specialized iterative optimization to effectively address noisy data challenges.

Figure 3 shows a visual comparison of the qualitative results of different WSCS methods on the Crack500, CFD, and DeepCrack datasets. In Figure 3, rows (1)–(3) are from the Crack500 testing set, rows (4)–(6) are from the CFD dataset, and rows (7)–(9) are from the DeepCrack dataset. From rows (1)–(3) of Figure 3, it can be observed that CrackCLIP is more robust to background noise on the Crack500 dataset and can accurately detect cracks even when the contrast between the cracks and the background is poor, as in row (1) of Figure 3. In addition, the three rows (4), (5), and (6) in the middle of Figure 3 show the visualization comparison on the CFD dataset. The cracks in the CFD dataset are mostly thinner and the contrast between the cracks and the background is low. In contrast to other models, which suffer from false detection and missed detection, CrackCLIP is more responsive to crack pixels. Rows (7)–(9) in Figure 3 show the prediction results of different methods on the DeepCrack dataset, which has a complex image background with varying crack scales. CrackCLIP demonstrates stable performance compared to other models. This can be attributed to the pre-trained CLIP model in CrackCLIP, which effectively exploits the semantic features of cracks. In summary, CrackCLIP not only achieves optimal accuracy on the Crack500 testing set but also demonstrates good generalization to complex crack scenarios in other datasets.

Experimental results on three publicly available datasets demonstrate that the CrackCLIP model outperforms most existing methods. Specifically, on the Crack500 dataset, the CrackCLIP model, which leverages vision-language alignment, exhibits excellent performance in handling crack image scenes with significant background noise interference. By utilizing the pre-trained CLIP model, only the parameters of four linear layers need to be fine-tuned to achieve effective crack segmentation. On the CFD and DeepCrack datasets, the CrackCLIP model demonstrates robust performance in detecting cracks with varying scales, textures, and thicknesses, particularly excelling in identifying thin cracks. In summary, the weakly supervised crack detection task is significantly improved by CrackCLIP, enhancing both the accuracy of crack prediction and the generalization capabilities in diverse crack scenarios.

### 4.6. Ablation Studies

**Figure 4:**
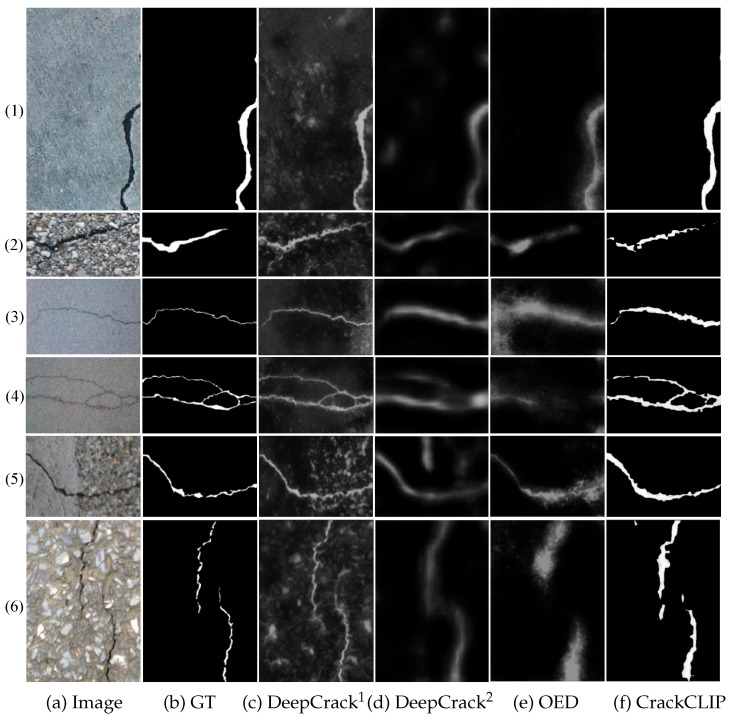
Visualization of crack prediction results for different crack segmentation backbones. Rows (1) and (2) are from the Crack500 test set, rows (3) and (4) are from the CFD dataset, and rows (5) and (6) are from the DeepCrack dataset.

**Figure 5:**
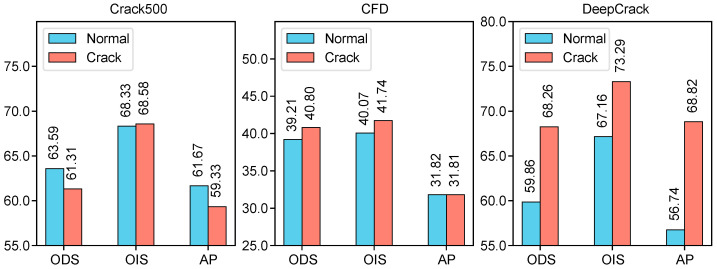
ODS, OIS, and AP values for CrackCLIP with different text prompts on the Crack500, CFD, and DeepCrack. “Normal” refers to normal defect text prompts, and “Crack” denotes specific crack text prompts.

To further validate the effectiveness of pseudo-label types, backbone networks, and crack-specific language prompts in CrackCLIP for weakly supervised crack segmentation, we conduct several analyses. We first examined the impact of different pseudo-label types on segmentation performance, with the results presented in Table 2. Next, we perform ablation studies on various backbone networks, with the results shown in Figure 4 and Table 3. Finally, we compare the experimental performance of general defect descriptions against specific crack descriptions, with the results illustrated in Figure 5. Through these analyses, we comprehensively demonstrated the contributions of each component to the model’s performance.

#### 4.6.1. Pseudo-Label Type

The generation of pseudo-labels is a fundamental component of weakly supervised learning, as it directly influences the quality of the final segmentation performance. To verify the effectiveness of different quality pseudo-labels for CrackCLIP, we use two different types of crack pixel-level pseudo-labels, including CAM-CRF [16] and CAM-Location [15]. CAM-CRF [16] is a pixel-level pseudo-label generated by a class activation map with CRF, while CAM-Location [15] is a pixel-level pseudo-label generated by class activation maps, crack patch location, and threshold segmentation combined to generate crack pixel-level pseudo-labels. Table 2 shows the quantitative prediction results of cracks for the CrackCLIP model using different types of pseudo-labels. As shown in Table 2, the fully supervised method (FSV) demonstrates the best performance across all datasets, which provides an upper bound for the performance of our weakly supervised methods, as FSV can utilize complete annotation information for training. However, with merely weak supervision, the performance of our proposed CrackCLIP approach is approaching the upper bound set by fully supervised methods on all three testing datasets. On the DeepCrack dataset, the CrackCLIP model using pseudo-labels CAM-Location outperforms the fully supervised approach, reduces model overfitting, and improves model generalization. CAM-CRF using CRF partially reduces background noise but does not eliminate it. The background noise may cause the pseudo-labels to contain incorrect positive samples, increasing the risk of model overfitting. In contrast, the pixel-level pseudo-labels generated by CAM-Location [15] eliminate the noise in the background as much as possible.

#### 4.6.2. Backbones

In order to evaluate the ability of the CLIP backbone for feature learning and generalization, we compare the weakly supervised crack segmentation results of CrackCLIP with those of several mainstream crack segmentation frameworks, including U-Net, DeepCrack^1^, DeepCrack^2^, and OED. Table 3 presents the performance of CrackCLIP with different backbone networks for image feature extraction on the Crack500, CFD, and DeepCrack datasets. The results indicate that CrackCLIP performs well on the Crack500 dataset compared to other network frameworks, achieving an OIS of 63.00%, ODS of 68.07%, and AP of 60.54%. To further validate the effectiveness and generalization of CrackCLIP, we conducted additional experimental analyses using the CFD and DeepCrack datasets. On the CFD dataset, CrackCLIP achieved an OIS of 39.15%, ODS of 39.82%, and AP of 32.01%. On the DeepCrack dataset, the OIS was 58.77%, ODS was 67.05%, and AP was 55.66%. These results demonstrate that CrackCLIP outperforms the other backbone networks on both the CFD and DeepCrack datasets.

Figure 4 presents a visualization of the qualitative results for each crack segmentation framework. CrackCLIP demonstrates superior visual performance compared to other models. In Figure 4, rows (1) and (2) are from the Crack500 test set, rows (3) and (4) are from the CFD dataset, and rows (5) and (6) are from the DeepCrack dataset. From Figure 4, it can be observed that all crack segmentation frameworks achieve better detection performance in scenes with low image background noise, wide cracks, and prominent structures. Additionally, it is evident in rows (2), (5), and(6) of Figure 4 that in cases of strong background noise, the contrast between the crack and the background is reduced, and the performance of other frameworks is more susceptible to interference from the noise, whereas CrackCLIP exhibits better robustness against such disturbances. This is primarily attributed to the fact that the CrackCLIP model employs a visual-language alignment-based approach, leveraging the strong visual-language alignment capabilities of the pre-trained CLIP model. The image encoder in the CLIP model, which is based on the Transformer architecture, effectively captures long-range dependencies in crack images and understands the global contextual information of cracks, thereby reducing the likelihood of background noise being incorrectly detected. However, from the visualization and quantification results of CrackCLIP in the last column of Figure 4, it is noted that the predicted crack width by the CrackCLIP model is broader, leading to an improvement in recall but a decrease in precision, meaning that background pixels surrounding the crack pixels are often incorrectly detected. From the quantitative metric AP, the AP value of CrackCLIP is lower compared to other methods. The possible reason for this is that during the image encoding process, the input image is divided into sequential image chunks, and the features of these extracted chunks are aligned with text features rather than individual pixels. Consequently, the edges of the cracks may not be as sharp as desired.

#### 4.6.3. Crack Text Prompts

We conducted an ablation study of crack language text prompts on three datasets: Crack500, CFD, and DeepCrack, to evaluate the effectiveness of our method. Figure 5 shows the generalization of normal defect text prompts versus specific crack text prompts in the weakly supervised crack segmentation task. In this study, we use generic text to express normal defect text prompts, such as “a photo of a {damaged {pavement}}”, while specific text prompts are designed to express crack text based on the apparent characteristics of cracks, such as “a photo of a {{pavement} with narrow break and opening}”. The proposed CrackCLIP is trained on the Crack500 dataset and tested on the Crack500 testing sets, CFD, and DeepCrack datasets. From Figure 5, it is evident that the CrackCLIP model using crack text prompts does not perform as well as the CrackCLIP model using normal text prompts on the Crack500 dataset. However, on the CFD and DeepCrack datasets, the CrackCLIP model using crack text prompts performs better. The experimental results indicate that normal text prompts help reduce the overfitting of CrackCLIP for the dataset of Crack500, while specific crack text prompts provide richer information as auxiliary supervisory signals for the crack text domain, thereby improving the generalization of the weakly supervised model for the datasets of CFD and DeepCrack.

## 5. Conclusions and Future Work

We propose CrackCLIP, a weakly supervised crack segmentation framework based on vision-language models. To achieve the alignment of crack images with text, we design specific text prompts that capture the apparent features and topology of cracks, enabling the learning of generalized crack semantic features and enhancing the generalization of crack detection. An additional linear layer is added to the image encoder module of the CLIP model to adaptively train the crack scene. The frozen pre-trained CLIP model provides a powerful feature representation for weakly supervised crack segmentation, allowing our approach to achieve better performance with reduced training costs. Furthermore, we evaluate the effectiveness of CrackCLIP on different crack datasets, demonstrating its robustness and versatility. By leveraging textual prompts to enhance the generalization of crack detection, we introduce a novel crack segmentation paradigm that offers innovative insights into this field. However, we also recognize limitations in our approach. Surface cracks are highly complex, and the limited nature of existing datasets may not adequately capture all variations across different scenarios. Additionally, weakly supervised information inherently introduces uncertainty. To address these challenges, we will collect more diverse training data from a wider range of scenarios and design more refined textual prompts. We will also explore finer-grained coarse segmentation methods to mitigate the uncertainty associated with weak supervision. We believe that these improvements will enable CrackCLIP to better address real-world challenges and advance research in this direction.

## Figures and Tables

**Table 1:** Evaluation of the segmentation results in ODS, OIS, and AP of different WSCS methods on the Crack500 testing set, CFD, DeepCrack (%).

Methods	Crack500			CFD			DeepCrack		
Metrics	ODS	OIS	AP	ODS	OIS	AP	ODS	OIS	AP
Grad-CAM [22]	53.12	56.86	49.89	23.16	17.52	14.07	44.88	52.42	37.33
PWSC [16]	56.64	63.73	**65.13**	8.56	14.46	7.72	37.05	43.95	44.31
GPLL [15]	45.04	56.69	45.46	18.74	19.41	14.88	65.97	73.19	72.28
CAC [19]	60.43	64.60	63.65	23.31	31.55	18.55	**71.01**	**77.98**	**75.51**
CrackCLIP	**61.31**	**68.58**	59.33	**40.80**	**41.74**	**31.81**	68.26	73.29	68.82

**Table 2:** The CrackCLIP segmentation results in ODS, OIS, and AP with different pseudo-label types were evaluated on the Crack500 testing set, CFD, and DeepCrack (%).

Methods	Pseudo-Label Types	Crack500			CFD			DeepCrack		
		ODS	OIS	AP	ODS	OIS	AP	ODS	OIS	AP
CrackCLIP	FSV	67.49	71.62	62.51	43.38	45.19	35.36	66.20	71.70	63.78
CrackCLIP	CAM-CRF [16]	**63.00**	68.07	**60.54**	39.15	39.82	**32.01**	58.77	67.05	55.66
CrackCLIP	CAM-Location [15]	61.31	**68.58**	59.33	**40.80**	**41.74**	31.81	**68.26**	**73.29**	**68.82**

**Table 3:** Evaluation of the segmentation results in ODS, OIS, and AP of different crack segmentation backbones on the Crack500 testing set, CFD, DeepCrack (%).

Methods	Crack500			CFD			DeepCrack		
Metrics	ODS	OIS	AP	ODS	OIS	AP	ODS	OIS	AP
U-Net [39]	55.88	63.20	**65.17**	9.09	16.74	9.58	44.17	54.32	**56.55**
DeepCrack^1^ [6]	56.64	63.73	65.13	8.56	14.46	7.72	37.05	43.95	44.31
DeepCrack^2^ [5]	57.93	64.44	62.23	20.18	26.25	14.79	44.57	49.61	48.89
OED [41]	52.01	61.14	54.02	10.24	15.89	4.43	26.16	32.71	9.42
CrackCLIP	**63.00**	**68.07**	60.54	**39.15**	**39.82**	**32.01**	**58.77**	**67.05**	55.66

## Data Availability

The data presented in this study are available on request from the corresponding author. Implementation of the proposed framework is publicly available on GitHub at the following link: https://github.com/liangfengjiao/CrackCLIP (accessed on 15 December 2024).
